# Retroperitoneoscopic adrenalectomy in an infant with adrenocortical virilizing tumor

**DOI:** 10.1590/S1516-31802002000300007

**Published:** 2002-05-02

**Authors:** Marcos Tobias-Machado, Jairo Cartum, Telma Murias Santos-Machado, Heloísa Amaral Gaspar, Alexandre Sibanto Simões, Ricardo Cruz, Renata Rodrigues, Roberto Vaz Juliano, Eric Roger Wroclawski

**Keywords:** Carcinoma, Laparoscopy, Adrenal, Adrenalectomy, Child, Virilization, Carcinoma, Laparoscopia, Adrenal, Adrenalectomia, Criança, Virilização

## Abstract

**CONTEXT:**

Adrenocortical virilizing tumors are rare in the pediatric age group. Laparoscopic surgery is the gold standard method for treatment of adrenal functional tumors under 6 cm in size, in adults. There has been ver y little use of laparoscopy in children and there is no report of its application in the treatment of adrenal carcinoma in childhood.

**DESIGN:**

Case report.

**CASE REPORT:**

We performed the first laparoscopic resection using retroperitoneal access for the treatment of an adrenocortical virilizing tumor in a pediatric patient. We believe that retroperitoneoscopic access is a viable and promising option for the treatment of adrenal tumors in children.

## RESUMO

**CONTEXTO:**

Tumores adrenocorticais virilizantes são raros na faixa etária pediátrica. A cirurgia laparoscópica é o método de escolha para o tratamento de tumores funcionantes de adrenal menores que 6 cm em adultos. O emprego da cirurgia laparoscópica para doenças da adrenal em crianças é pouco freqüente e não existe relato de sua aplicação no tratamento de tumores adrenocorticais na infância.

**TIPO DE ESTUDO:**

Relato de caso.

**RELATO DE CASO:**

Nós realizamos a primeira ressecção laparoscópica utilizando o acesso retroperitoneal para o tratamento de tumor adrenocortical virilizante em paciente pediátrico. Acreditamos que o acesso retroperitoneoscópico é viável, representando opção promissora para o tratamento de tumores adrenais na infância.

## INTRODUCTION

Adrenocortical tumors are generally rare in childhood, but more frequent in adults. Curiously, the prevalence of these tumors seems to be higher in the southern hemisphere.

A recent and extensive review of adreno-cortical tumors in children revealed that, because it is rarely diagnosed, no cases of laparoscopic adrenalectomy have been described in the treatment of adrenal carcinoma in childhood.^1^

The objective of this study is to report on the first case of laparoscopic retroperitoneal adrenalectomy performed in a oneand-a-half-year-old child who presented a 5-cm tumor in the right adrenal gland, as well as clinical signs of virilization.

## CASE REPORT

The patient was a one-and-a-half-year-old child presenting clinical signs of virilization that were characterized by increased penile volume, enlargement of the testicles and pubic hair growth over a period of three months.

The laboratory tests showed:

- Testosterone: 341.30 ng/dl (< 30 ng/dl)
- Follicle Stimulating Hormone: 4.2 mU/ml (4-25 mU/ml)
- Luteinizing Hormone: 3.8 mU/ml (1-8 mU/ml)
- Androstenedione: 8.8 nmol/l (<1.75 nmol/l)
- Hydroxyprogesterone: 10.6 ng/ml (0.5-2.5 ng/ml)
- Dehydroepiandrosterone sulfate: 1070 nmol/l (69-519 nmol/l)
- Cortisol: 256 nmol/l (149-690 nmol/l)

The abdominal ultrasound examination revealed a localized 5-cm tumor in the right adrenal gland. The staging was negative for distant metastases.

**Figure 1:**
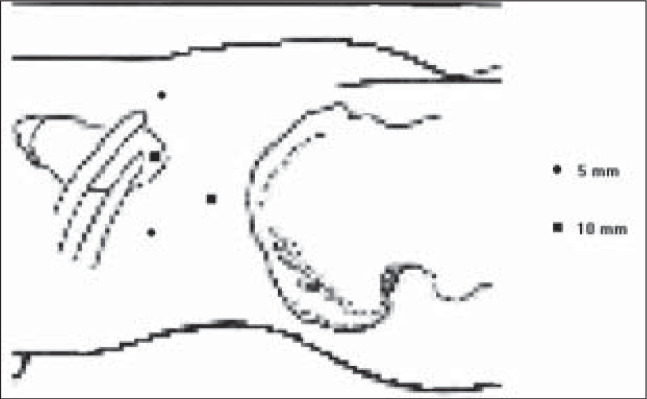
Ports configuration used in the procedure.

The diagnosis of an adrenocortical right functional tumor led to the choice of laparoscopic retroperitoneal adrenalectomy by means of 4 ports (Figure 1).

**Figure 2:**
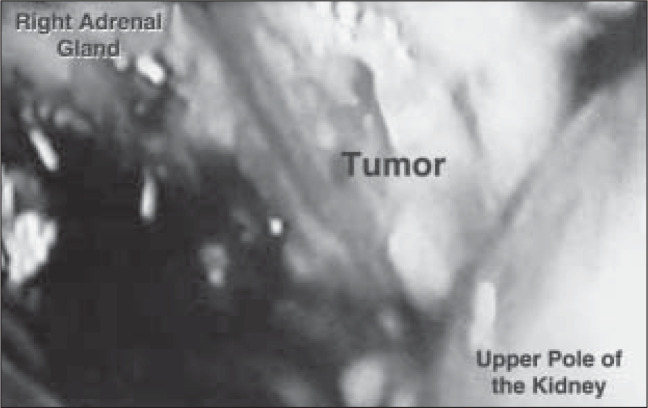
Extraperitoneal laparoscopic vision.

Digital dissection was used to create the area to perform the procedure and the retroperitoneal air pressure was determined as 12 mmHg. Gerota's fascia was opened, with subsequent dissection of the upper rhombus, yielding the identification of the adrenal tumor (Figure 2).

The inferior vena cava was identified medially, with control of the adrenal vessels obtained by cauterization and application of metallic clips.

The specimen was removed in a laparoscopic bag, through a small widening of one of the 10-mm incisions. The duration of the surgery was two hours and the estimated blood loss was 50 ml.

The patient presented a favorable surgical evolution. Bronchopneumonia developed from the 3^rd^ postoperative day onwards, and the patient was only discharged from the post-surgical ward on the 14^th^ postoperative day, because of the pneumonia.

**Figure 3:**
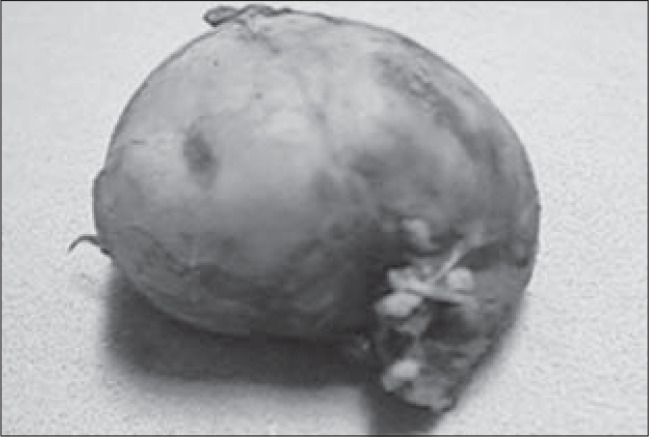
Macroscopic appearance of the tumor. It is 5 cm in diameter and has precise limits and a weight of 50 g.

Surgical pathology examinations yielded a diagnosis of cortical adrenal carcinoma with capsular involvement and free margins. The tumor weighed 50 grams (Figure 3).

The hormone levels were normal when determined at the one-year postoperative follow-up, without any evidence of residual tumor.

## DISCUSSION

Adrenal cortex tumors are considered to be rare in childhood, totaling between 0.3 and 0.4% of the solid tumors in children. Virilization is the most common sign presented.^1^

Ultrasound is the imaging method of choice for defining the diagnosis and the therapeutic options. It is especially useful in children, because it adequately identifies tumors larger than 2.5 cm and excludes the need for additional examinations that require anesthesia.

Surgical resection is the best curative management.

In adults, laparoscopic surgery has become the method of choice for treating lesions of less than 6 cm in size, because it allows for faster recovery, in addition to having a better cosmetic outcome.^2^

Most authors have chosen transperitoneal access, as this makes it possible to have a larger working area, with better visibility and better conditions for identifying the anatomical parameters. The extraperitoneal approach, however, is an option that permits quick access to the retroperitoneal organs, without the need for extensive dissections.

Valla et al. reported on 88 laparoscopic surgeries in children, of which only two were adrenalectomy. Neither of them was due to the presence of an adrenocortical tumor.^3^ Other authors have reported the use of the laparoscopic approach in children with pheochromocytomas.^4^ After an extensive review of the literature, we have come to believe that ours is the first surgery to use the laparoscopic retroperitoneal approach for the resection of an adrenocortical virilizing tumor.^1^

The authors propose that this approach is technically viable, under conditions of limited operational space and peritoneal thinning. Conversely, the small amount of fat tissue facilitates extraperitoneal dissection and localization of the adrenal gland. The duration of the surgery was reasonable, matching that obtained in open surgery. Early discharge did not take place in this case, due to the specific clinical complication.

Laparoscopic retroperitoneal access for the surgical treatment of the adrenal gland constitutes an attractive option for tumors of less than 5 cm in size, including localized neuroblastomas without spontaneous regression, virilizing tumors and pheochromocytomas. The authors believe that laparoscopic surgery is a promising option in selected cases, for the treatment of children with localized adrenal tumors that do not show invasion of neighboring structures.
